# An evaluation of information online on artificial intelligence in medical imaging

**DOI:** 10.1186/s13244-022-01209-4

**Published:** 2022-04-25

**Authors:** Philip Mulryan, Naomi Ni Chleirigh, Alexander T. O’Mahony, Claire Crowley, David Ryan, Patrick McLaughlin, Mark McEntee, Michael Maher, Owen J. O’Connor

**Affiliations:** 1 Cork University Hospital/Mercy University Hospital, Cork, Ireland; 2 University College Cork, Cork, Ireland; 3 Cork University Hospital, Cork, Ireland; 4 South Infirmary Victoria University Hospital, Cork, Ireland

**Keywords:** Artificial intelligence in radiology, Perspectives on evolution of radiology, Future impact on the radiologist, Radiology recruitment, Radiology efficiency

## Abstract

**Background:**

Opinions seem somewhat divided when considering the effect of artificial intelligence (AI) on medical imaging. The aim of this study was to characterise viewpoints presented online relating to the impact of AI on the field of radiology and to assess who is engaging in this discourse.

**Methods:**

Two search methods were used to identify online information relating to AI and radiology. Firstly, 34 terms were searched using Google and the first two pages of results for each term were evaluated. Secondly, a Rich Search Site (RSS) feed evaluated incidental information over 3 weeks. Webpages were evaluated and categorized as having a positive, negative, balanced, or neutral viewpoint based on study criteria.

**Results:**

Of the 680 webpages identified using the Google search engine, 248 were deemed relevant and accessible. 43.2% had a positive viewpoint, 38.3% a balanced viewpoint, 15.3% a neutral viewpoint, and 3.2% a negative viewpoint. Peer-reviewed journals represented the most common webpage source (48%), followed by media (29%), commercial sources (12%), and educational sources (8%). Commercial webpages had the highest proportion of positive viewpoints (66%). Radiologists were identified as the most common author group (38.9%). The RSS feed identified 177 posts of which were relevant and accessible. 86% of posts were of media origin expressing positive viewpoints (64%).

**Conclusion:**

The overall opinion of the impact of AI on radiology presented online is a positive one. Consistency across a range of sources and author groups exists. Radiologists were significant contributors to this online discussion and the results may impact future recruitment.

**Supplementary Information:**

The online version contains supplementary material available at 10.1186/s13244-022-01209-4.

## Keypoints

- Consensus?
- An overall positive opinion exists online towards AI on the future of radiology.
- Radiologists?
- A high proportion of radiologists believe there will be a positive impact.

## Background

Artificial intelligence (AI) involves the use of computer algorithms to perform tasks typically associated with human intelligence [1]. The role of AI in medical imaging has progressed to various stages of development, application and refinement over the past 10–15 years. Consequentially, publications on AI in medical imaging have exponentially increased from about 100–150 per year in 2007–2008 to 700–800 per year in 2016–2017 [2]. Several studies pertaining to dermatology, pathology, and ophthalmology have shown the potential and clinical utility of AI algorithms. For example, skin cancer, the most diagnosed malignancy worldwide, is primarily diagnosed visually. Deep neural networks (DNN) have demonstrated equivalence with consultant dermatologist diagnostic ability [3]. Hence the early evolution of AI has leaned towards the visual sciences and its application to radiology an extension of this.

Medical imaging interpretation requires accuracy, precision, and fidelity. At its essence it is a visual science whereby the interpreter translates either a single or series of images into a succinct report to answer a clinical question and guide evidence-based management. Studies report that on average a radiologist must interpret one image every 3–4 s in an 8-h workday to meet workload demands [4] and with the compound annual growth rate (CAGR) of diagnostic imaging estimated to be 5.4% until 2027 [5] increasing workloads are expected. Burnout has been ubiquitously reported among medical specialties (~ 25–60%) [6, 7] with limited solutions being proposed and implemented; thus many key advantages may be conferred by the incorporation of AI into radiological practice. The applications of AI in radiology can be broadly divided into diagnostic and logistic. Computer-aided diagnostics (CAD) may facilitate earlier detection of abnormalities, improve patient outcomes, reduce medico-legal exposure, and decrease radiologist workload. Logistic improvements would include optimization of workflow, prompt communication of critical findings and more efficient vetting and triage systems.

Historically, apprehension has existed concerning recruitment within the medical and radiological community as a result of AI. Focused assessment of individual stakeholder groups in relation to AI in radiology demonstrated a wide spectrum of opinion. Studies of medical student perspectives in North America, Europe and the United Kingdom conveyed heterogenous opinions on the potential implications of AI on radiology possibly with geographical variation [8–10]. A recurrent theme in early studies is the large educational gap in medical schools regarding the capability, utility, and limitations of AI. A European multi-centre study of both radiologists in training and consultants performed in France [11] demonstrated an overall positive perspective; however, a majority expressed concerns regarding insufficient information on AI and its potential implications. Ten years ago, the end of radiology as a career was being heralded. Hence radiology residency applications reduced in response to concerns about the future of radiology as a career [12]. Ten years ago, perception probably reflected local concerns in the absence of experience. It has been shown that more positive opinions have been expressed by those medical students with exposure to AI and radiology [9]. The transition from discourse about the potential of AI to its integration and use should have modified opinions based on practice and experience.

Therefore, this paper aimed to quantify the proportion of positive, negative, balanced, and neutral viewpoints presented on the internet in relation to the impact of AI on radiology. The purpose of this was to determine the global and regional perception of AI in radiology, and thus, conclude as to where the future of radiology may lie.

## Methods

### Data collection

Two search methods were used to evaluate information online relating to artificial intelligence in medical imaging. The first search method screened existing data on AI in radiology at the time of search. The second method identified a live stream of articles relating to AI as they were released on the web. Searches were carried out independently by two of the investigators.

Thirty-four key search phrases were established (Additional file 1: Appendix 1). Phrases were generated with input from a medical student, healthcare professional, non-consultant hospital doctor and prospective radiology trainee. These phrases were than validated by two consultant radiologists (M.M.M., O.J.O’C). The phrases were chosen to reflect a broad range of search terms encompassing a multidisciplinary opinion to the impact of AI on the radiology service.

### Data identification

#### Existing data

This search was performed on ‘All’ content in the Google search engine and was conducted over the period 25th January 2021–7th February 2021. The google search engine has over 90% of the market share and thus was felt to be reasonably representative of the population on a global scale [13]. The google search was performed for the 34 key phrases in an identical manner. Results were limited to the English language and open access academia or where no financial stipulation was required to access the article. We reviewed the first two pages of Google results for these searches, as numerous studies on user behaviour have indicated that 95% of users choose websites listed on the first page of results, leaving only 5% reviewing results on any subsequent page [14–18]. While date of publication was not a selection criterion all included articles from the google search were created within the past 5 years.

#### Live stream

A Rich Site Summary (RSS) feed search strategy was used to evaluate the written incident information over a 3-week period (07/03/21–28/03/21) as a surrogate for postings on news media and social media. The same 34 key phrases were entered into Google Alerts. This provided a continuous search for new relevant online content appearing subsequently. This content was then analyzed and organized appropriately.

#### Data sourcing

The source of each relevant post was identified. The source website was then assigned a sub-type based on the ‘About Us’ section. The source subtypes were segregated as either journal, media, commercial, education or other if outside of these categories. For published academia, it was noted whether it was from a peer- reviewed and/or indexed journal (PUBMED). Where an identifiable author existed, it was subtyped into radiologist, journalist, non-radiologist doctor, radiographer and other. The geographical origin and date of issue was also noted, where available.

### Data categorization

The web pages identified by the dichotomized search strategy were analyzed by each investigator homogenously. Firstly, all Google advertisements were omitted. Each post was then categorized as either relevant or non-relevant. Non-relevant posts included those failing to provide information on AI in medical imaging (such as a journal calling for abstracts/submissions) or academia related posts that were not open access, duplicate posts or posts that were inaccessible.

Relevant posts were divided as either having an overall positive, negative, balanced, or neutral viewpoint. The assessment and categorization of this information was carried out by two senior authors (M.M.M., O.J.O.C), both of whom are academic consultant radiologists working in a large teaching hospital. The assessment was done in tandem, and the final decision was arrived at by consensus.

### Relevance

#### Positive

Positive viewpoints were themed as changes brought about by AI which would result in increased employment, service expansion, efficiency, fidelity of interpretation, improved patient care, better quality assurance and more job satisfaction. Additional file 1: Appendix 2 provides a sample of positive viewpoints as extracted from the data of included posts. Webpages that contained predominantly positive information and concluded with an overriding positive viewpoint were categorized as ‘Positive’.

#### Negative

Negative viewpoints were those that displayed a contrary theme to the positive *viewpoint* (see Additional file 1: Appendix 2).

#### Balanced and neutral

Webpages categorized as ‘Balanced’ listed comparable amounts of positive and negative points without giving an overall positive or negative viewpoint. Webpages categorized as ‘Neutral’ objectively presented information relating to artificial intelligence and radiology but did not discuss how this would impact, be it negatively or positively, on the field of radiology. The fundamental difference between the ‘Balanced’ and ‘Neutral’ categories is that balanced webpages explicitly discussed how aspects of artificial intelligence would impact the field of radiology while neutral webpages did not (see Additional file 1: Appendix 2).

### Data analysis

Data compilation and statistical analyses were performed using Microsoft Excel (Microsoft Corporation, Redmond, Washington, USA) and Google Sheets (1600 Amphitheatre Parkway, Mountain View, California, United States). Descriptive statistics were used to summarize data. Frequency analyses were performed for categorical variables.

## Results

A total of 680 Google pages relating to AI in medical imaging were identified. Of these, 561 pages were deemed relevant and accessible. Duplicate pages were removed, leaving 248 pages for evaluation.

**Fig. 1:**
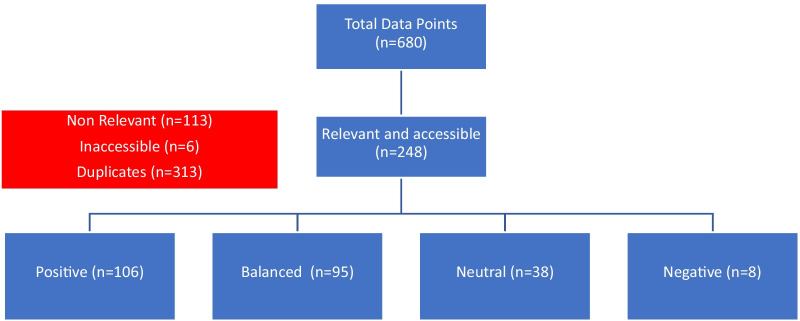
Schematic of google search and results summary

Forty-three percent (*n* = 106) of these pages expressed the overall view that AI would have a positive impact on the radiologist and the radiology department; 3.2% (*n* = 8) presented an overall negative viewpoint; 38.2% (*n* = 95) presented a balanced viewpoint and 15.3% (*n* = 38) presented a neutral viewpoint (see Fig. 1).

Forty-eight percent (*n* = 120) of the relevant pages were from open-access peer-reviewed journals; 30.2% (*n* = 75) were from media sources; 12.9% (*n* = 32) from commercial websites and 8.5% (*n* = 21) from educational sources. Table 1. Summarises the allocated categories of origin and viewpoint conveyed. The type of media source along with the details of specific commercial company can be seen in Additional file 1: Appendix 3.1 & 3.2. Commercial web pages had the highest proportion of positive viewpoints i.e., 66%, followed by media web pages at 52%, peer-reviewed journals at 37% and educational web pages at 14%. On the other hand, media web pages had the greatest proportion of negative viewpoints at 5%, followed by peer-reviewed journals at 3%. Negative viewpoints were not identified among commercial, educational, or other sources. Peer-reviewed journals had the greatest proportion of balanced viewpoints at 48%, while educational web pages had the greatest proportion of neutral viewpoints at 43%.

**Table 1:** Summary of categorization of posts by origin with percentage

*n* = 248	Journal		Media		Commercial		Education	
	*n* = 120	48.39%	*n* = 75	30.20%	*n* = 32	12.90%	*n* = 21	8.47%
Positive	44	36.67%	39	52.00%	21	65.63%	3	14.29%
Negative	4	3.33%	4	5.33%	0	0.00%	0	0.00%
Balanced	58	48.33%	24	32.00%	4	12.50%	9	42.86%
Neutral	14	11.67%	8	10.67%	7	21.88%	9	42.86%

An identifiable named author was displayed on 93% (*n* = 230) of web pages, with radiologists responsible for 38.7% (*n* = 89); journalists represented 20% of authors (*n* = 46); doctors working in other specialties represented 6.9% (*n* = 16); and radiographers represented 4.8% (*n* = 11). Other authors not falling into the aforementioned categories made up the remaining 29.6% (*n* = 68). Researchers, lawyers, and marketing managers were amongst those in the ‘Other’ category.

**Fig. 2:**
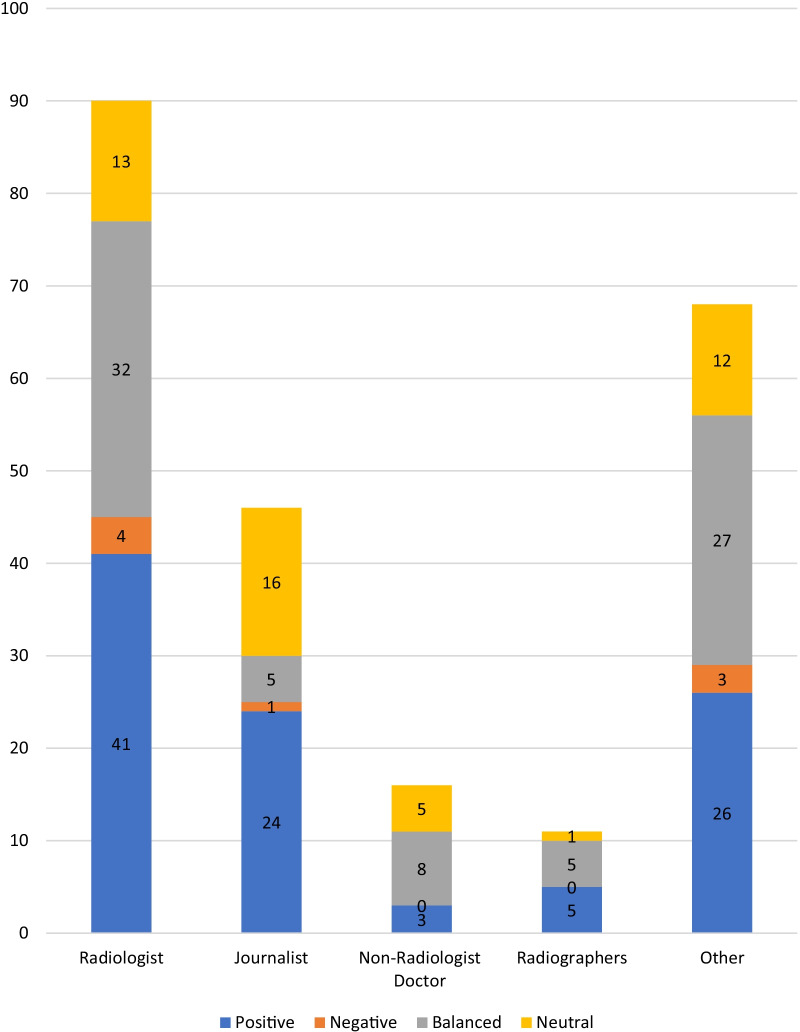
Number of overall viewpoints presented by each author group. *N* = 230

Web pages authored by journalists had the highest percentage of overall positive viewpoints (52%, *n* = 24). This was followed by web pages authored by radiologists (46%, *n* = 41) and radiographers (45%, *n* = 5). Web pages authored by non-radiologist doctors accounted for the lowest proportion of positive viewpoints (18.8%, *n* = 3). Four percent of web pages authored by radiologists (*n* = 4) or by those falling into the ‘Other’ category (*n* = 3) had negative viewpoints, followed by web pages authored by journalists at 2% (*n* = 1). There were no negative viewpoints identified in web pages authored by radiographers or non-radiologist doctors. Those authors falling into the category “Other” had the highest proportion of balanced viewpoints at 39.7% (*n* = 27), while journalists had the greatest proportion of neutral viewpoints at 34.7% (*n* = 16). See Additional file 1: Appendix 4.1 for tabulated summary (Fig. 2).

**Fig. 3:**
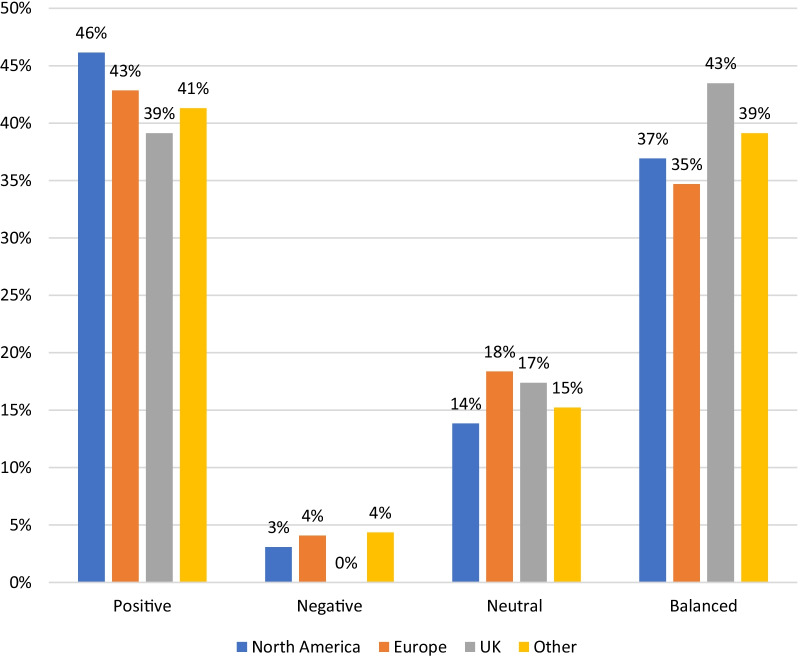
Geographical origin of viewpoint (percentage)

There were 130 pages authored in North America expressing 60 positive, 48 balanced, 18 neutral and 4 negative pages of content. In Europe (*n* = 49), there were 21 positive, 17 balanced, 9 neutral and 9 negative pages authored. The United Kingdom had the greatest number of European authored pages, and these expressed 9 positive, 10 balanced, 4 neutral and 0 negative opinions (*n* = 23). The distribution of the remaining pages from Europe was as follows: Netherlands—6, Germany—6, Italy—8, Ireland—4, Belgium—5, Norway—1, Denmark—1, Switzerland—5, Austria—1, Cyprus—3, Europe not specified—9. Finally, a miscellaneous group including: Australia—11; Israel—4; Asia—12; South America—2, Africa—2; and Not available—14, expressed 19 positive, 18 balanced, 7 neutral and 2 negative opinions in the pages that were authored. This frequency data is presented in Table 2 with corresponding percentages in Fig. 3.

**Table 2:** Geographical origin of viewpoints

	Number	Positive	Negative	Neutral	Balanced
*Geographical origin*					
North America	130	60	4	18	48
Europe	49	21	2	9	17
UK	23	9	0	4	10
Other	46	19	2	7	18

**Fig. 4:**
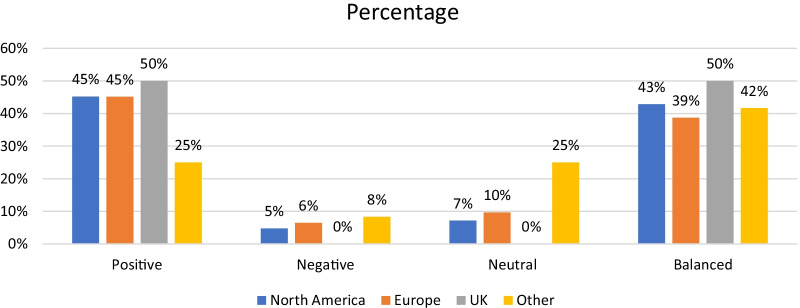
Geographical origin and radiologist viewpoint percentage

Radiologists in North America (*n* = 42) authored 19 positive, 18 balanced, 3 neutral and 2 negative viewpoints. In Europe, radiologists (*n* = 31) authored 14 positive, 12 balanced, 3 neutral and 2 negative viewpoints. UK radiologists authored four pages expressing two positive and two balanced perspectives. These data are presented in Table 3 and Fig. 4.

**Table 3:** Geographical origin of radiologist and viewpoint

Origin	Number	Positive	Negative	Neutral	Balanced
North America	42	19	2	3	18
Europe	31	14	2	3	12
UK	4	2	0	0	2
Other	12	3	1	3	5

**Fig. 5:**
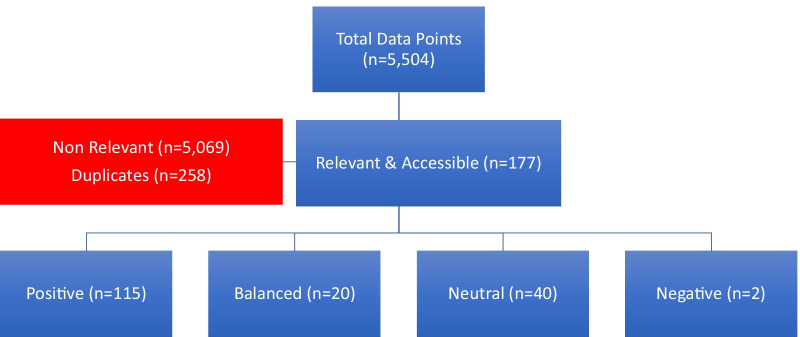
Schematic of live Google Alert RSS feed and results summary

The Google Alerts RSS feed identified 5504 new posts over the 3-week period from 34 search terms. Of the alerts identified, 177 were deemed relevant and accessible. Sixty-five percent (*n* = 115) of the posts expressed an overall positive viewpoint; 11% (*n* = 20) a balanced viewpoint; 23% (*n* = 40) a neutral viewpoint; and 1% (*n* = 2) an overall negative viewpoint towards the potential impact of AI on radiology (Fig. 5).

Of the relevant posts, the majority were of media origin (86%, *n* = 152); peer-reviewed journals accounted for 8% (*n* = 14); 4% (*n* = 7) were from commercial websites; and 2.3% (*n* = 4) were from other sources. Commercial webpages had the highest percentage of overall positive viewpoints (85.7%, *n* = 6). This was followed by media webpages (67%, *n* = 102), peer-reviewed journals (35.7%, *n* = 5), and webpages that fell under the category ‘other’ (25%, *n* = 1). Forums, educational webpages, and blogs composed the ‘other’ category. Peer-reviewed journals had the greatest percentage of balanced viewpoints (21.4%, *n* = 3), followed by those that fell under the category ‘other’ (25%, *n* = 1). One (7%) article from a peer-reviewed journal had an overall negative viewpoint, as did one (0.66%) of the media webpages. No negative viewpoints were identified in the commercial category. See Table 4 for summary.

**Table 4:** Summary of categorization of posts by origin with percentage

*n* = 177	Journal		Media		Commercial		Other	
	14	7.91%	152	85.88%	7	3.95%	4	2.26%
Positive	5	35.71%	102	67.11%	6	85.71%	1	25.00%
Negative	1	7.14%	1	0.66%	0	0.00%	1	25.00%
Balanced	3	21.43%	13	8.55%	0	0.00%	1	25.00%
Neutral	5	35.71%	36	23.68%	1	14.29%	1	25.00%

**Fig. 6:**
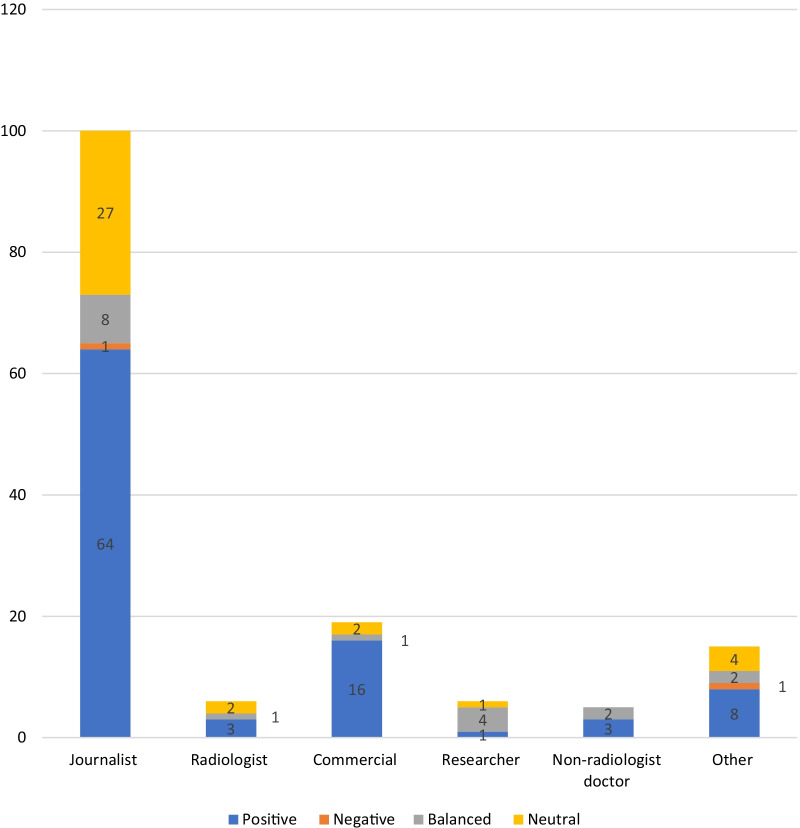
Number of overall viewpoints presented by each author group. *N* = 151

An identifiable named author was present on 85% (*n* = 151) of the relevant webpages identified by the Google Alerts RSS feed. The majority of listed authors were journalists (66%, *n* = 100). This was followed by commercial authors (12.6%, *n* = 19), radiologists (4%, *n* = 6), researchers 4% (*n* = 6), and doctors working in other specialties 3.3% (*n* = 5). Other authors not falling into the categories represented 9.9% (*n* = 15) of the contributors. This is illustrated in Fig. 4. Marketing managers, media editors, and students were amongst those that made up the ‘other’ category. Webpages with a commercial author had the highest percentage of overall positive viewpoints 84% (*n* = 16). This was followed by webpages authored by journalists 64% (*n* = 64); non-radiologist doctors 60% (*n* = 3); ‘other’ authors 53% (*n* = 8); and radiologists 50% (*n* = 3). Researchers had the greatest percentage of balanced viewpoints 67% (*n* = 4), while radiologists had the greatest percentage of neutral viewpoints 33% (*n* = 2). One webpage authored by a journalist (1%) and one authored by an author in the ‘other’ category (7%) had overall negative viewpoints. This data summarized and tabulated can be seen in Additional file 1: Appendix 4.2 (Fig. 6).

## Discussion

Opinions and forecasts concerning the role and impact of AI on medical imaging have exploded in last number of years primarily due to recent advancements in AI products for radiology. These viewpoints can be positive, negative, balanced, or neutral in their content. AI in medical imaging was first mentioned in the literature in the 1950’s and has evolved substantially since the early 2000’s with the advent of machine learning (ML) and deep learning (DL) algorithms [19]. The number of AI exhibitors at the annual meeting of the Radiological Society of North America (RSNA) and the European Congress of Radiology (ECR) has tripled from 2017 to 2019 [20, 21]. Since 2016, the US Food and Drugs administration (FDA) has approved 64 AI ML-based medical imaging technologies with 21 of these specializing in the field of Radiology [22]. In Europe, 240 AI/ML devices have been approved over the 2015–2020 period by the Conformité Européene (CE) with 53% for use in radiology [23]. In 2019, The European Society of Radiology published a white paper to provide the radiology community with information on AI and a further study by the ESR demonstrated that there is a demand amongst the radiological community to integrate AI education into radiology curricula and training programs including issues related to ethics legislation and data management [24]. The aim of the present paper was use internet activity to determine current opinion on whether AI is a threat or opportunity to the field as this will have impact on recruitment and resource allocation to radiology.

We observed that a wide diversity of commentators were engaged dialog pertaining to AI in radiology ranging from those with professional and academic backgrounds to those with individual and organizational interests. While these authors predictably included healthcare professionals, there was also a significant representation from those with media and commercial backgrounds. Opinions on AI in radiology were therefore gathered from authors with a wide variety of occupations and backgrounds including radiologists, non-radiology physicians, journalists, researchers, radiographers, commercial managers, physicists, lawyers, computer scientist, data officers, engineers’, students, and pharmacists. There was a relatively equal division of authorship between North America and Europe. This distribution was also demonstrated among radiologist authored pages included in this study. This professional and geographic diversity of authors provides a more complete and international sample of opinions on the impact of AI on radiology.

Radiologists repeatedly expressed the opinion that inclusion of AI algorithms could help with labour intensive tasks, improve efficiency and workflow. They also opined against the potential of AI replacing radiologists. Numerous studies in the literature also argued against AI replacing radiologists [25, 26]. An example of two comments made by radiologists included:

The higher efficiency provided by AI will allow radiologists to perform more value-added tasks, becoming more visible to patients and playing a vital role in multidisciplinary clinical teams

And

Radiologists, the physicians who were on the forefront of the digital era in medicine, can now guide the introduction of AI in healthcare - The time to work for and with AI in radiology is now

Radiographers expressed the opinion that utilizing AI algorithms could:

ultimately lead to a reduction in the radiation exposure while maintaining the high quality of medical images

and that radiographers would be vital in building quality imaging biobanks for AI data bases. Interestingly, radiographers also wrote that AI should be integrated into the medical radiation practice curriculum and there should be more emphasis on radiomics. Furthermore, radiographers expressed the belief that emotional intelligence not artificial intelligence is the cornerstone of all patient care and while the concept of ‘will a robot take my job’ may be a hot topic, they believe that patient’s will not accept their radiographs being taken by a robotic device.

This study identified a total of ten negative viewpoints which included comments from radiologists—5, a lawyer—1, a journalist—1 and a neuroscience Ph.D. student—1. Examples include:

In the long-term future, I think that computers will take over the work of image interpretation from humans, just as computers or machines have taken over so many tasks in our lives. The question is, how quickly will this happen?

And

Radiologists know that supporting research into AI and advocating for its adoption in clinical settings could diminish their employment opportunities and reduce respect for their profession. This provides an incentive to oppose AI in various ways

And

An artificially intelligent computer program can now diagnose skin cancer more accurately than a board-certified dermatologist and better yet, the program can do it faster and more efficiently

And

A.I. is replacing doctors in fields such as interpreting X-rays and scans, performing diagnoses of patients’ symptoms, in what can be described as a ‘consulting physician’ basis

A recent editorial in the Radiological Society of North America (RSNA) highlighted a number of high-profile negative viewpoints made a number of years ago relating to the impact of AI on radiologists [27]. This included an AI pioneer who was recently awarded the Association for Computing Machinery Turing Award, “We should stop training radiologists now” [27]. Secondly a venture capitalist, Vinod Khsla proclaimed in 2017 ‘that the role of the radiologist will be obsolete in 5 years’ and replaced with ‘sophisticated algorithms’ [28] and furthermore an American ‘Affordable Care’ architect remarked at the 2016 American College of Radiology Annual meeting that radiologists will be replaced by computer technology in 4–5 years [29] and that ‘in a few years there may be no specialty called radiology’[30].

Interestingly, many of the opinions regarding timeframes during which AI were predicted to replace radiologists have already expired with a relatively minor uptake of AI in imaging interpretation and without signs of AI replacing radiologists at present. These controversial viewpoints have potential to grab headlines but are not without potential for negative impact on the future of radiology and particularly on recruitment of future radiologists, given that studies have shown that medical students are less likely to consider pursuing a career in radiology because of the apparent threat of AI to the specialty [8–10, 31].

This study found that the overwhelming majority of web pages assessed had favourable viewpoints with very few negative viewpoints identified. This finding is consistent with a recent social media-based study showing that discussions around AI and radiology were astoundingly positive, with an increasing frequency of positive discussions identified over a 1-year period [32]. Taken together, these findings suggest a shift in opinion from a once negative view to a more positive one.

Of the webpages identified using the Google search engine, Radiologists were found to be the most common author group, making up 38.5% of all identifiable authors. These webpages were predominantly peer-reviewed journal papers and media articles. These findings highlight that radiologists are actively involved in both AI-related research and online discussions relating to AI and the field of radiology. Radiologists have been encouraged to play an active role in the development of applications of AI in medical imaging to ensure appropriate implementation and validation of AI in clinical practice [26, 33]. In 2017, The American College of Radiology established The Data Science Institute partly with this purpose in mind [34].

The main limitation of this study was the use of subjective assessment to qualify information into positive, negative, neutral, and balanced. This introduces potential for observer bias in determining the overall viewpoint of posts, but it was attempted to minimize this by using two senior radiologists as the assessors. We did not quantitatively assess readability of posts. We only used one search engine ‘Google’ and limited to just the English language and to the first two pages of each search term, a strategy following previous publications and backed by behavioral studies which have indicated that 95% of users choose websites listed on the first page of results, leaving only 5% reviewing results on any subsequent pages.

We acknowledge that the list of search terms in Additional file 1: Appendix 1 is not exhaustive and is just a representative sample of the actual terms that may be used when searching for AI in medical imaging but by using a broad range of terms and studying the first two pages of findings that these search results would yield the most relevant information. The RSS feed was used as a surrogate for incident information and may not be wholly representative of information found in social media news feeds, Twitter, and other sites. There is also potential that a single 3-week alert period may be biased by news and media events that occurred during that time.

In Conclusion, authors of 43% of all pages evaluated expressed the overall opinion that AI would have a positive impact on the radiologist and the radiology department; 38.3% presented a balanced viewpoint; 15.3% presented a neutral viewpoint; and 3.2% presented a negative viewpoint. We have demonstrated that the overall view presented online is a positive one that AI will benefit the specialty. We should be excited and look forward to advancements in this technology which has the potential to improve accuracy of diagnosis in diagnostic radiology, reduce errors and improve efficiency in dealing with rapidly increasing workloads.

## Supplementary Information

**Additional file 1:** 34 key search phrases used in both static Google search and Rich Site Summary feed search strategy.

## Data Availability

The datasets used and/or analyzed during the current study are available from the corresponding author on reasonable request.

## Abbreviations

AI: Artificial intelligence
CAGR: Compound annual growth rate
CAD: Computer-aided diagnostics
DL: Deep learning
ECR: European congress of radiology
FDA: Food and drug agency
ML: Machine learning
RSS: Rich site summary
RSNA: Radiological society of North America
