# Supplementary material for: An evaluation of information online on artificial intelligence in medical imaging
Source: Insights Imaging. 2022 Apr 25;13:79. doi: 10.1186/s13244-022-01209-4 (PMC9038977; doi:10.1186/s13244-022-01209-4)
Supplement: Supplementary file 1 — Additional file 1: 34 key search phrases used in both static Google search and Rich Site Summary feed search strategy. [file 13244_2022_1209_MOESM1_ESM.docx]

# appendix 1.

## Search terms

• Artificial intelligence in radiology

• Artificial intelligence in diagnostic imaging

• Artificial intelligence in medical imaging

• Augmented intelligence in radiology

• Augmented intelligence in Diagnostic Imaging

• Augmented intelligence in medical imaging

• Opinions of AI in radiology

• Opinions of AI in diagnostic imaging

• Opinions of AI in Medical imaging

• Clinical applications of AI in radiology

• Clinical applications of AI in diagnostic imaging

• Clinical applications of AI in medical imaging

• AI applications in diagnostic imaging

• Deep learning applications radiology

• Deep learning applications diagnostic imaging

• Deep learning applications medical imaging

• Machine learning applications radiology

• Machine learning applications diagnostic imaging

• Machine learning applications medical imaging

• Medical imaging informatics

• Impact of Artificial intelligence on radiology

• Impact of Artificial intelligence on diagnostic imaging

• Impact of Artificial intelligence on medical imaging

• Impact of Artificial intelligence on radiologists

• Impact of Artificial intelligence on radiographer

• Ethical considerations of AI in radiology

• Ethical considerations of AI in diagnostic imaging

• Ethical considerations of AI in medical imaging

• Medical-legal considerations of AI in radiology

• Medical-legal considerations of AI in diagnostic imaging

• Medical-legal considerations of AI in medical imaging

• Opportunities for AI in radiology

• Opportunities for AI in diagnostic imaging

• Opportunities for AI in medical imaging

# appendix 2.

## Examples of Viewpoints

Positive viewpoints:

- ‘Working time saved by AI will increase the quantity and quality of clinical communication with patients and clinicians’
- ‘Job positions will increase as a result of AI’
- ‘The role of the radiologist will expand’...
- ‘AI will make radiologists more sub- specialty focused’...
- ‘More accurate and reproducible radiological assessments can be made’....
- ‘AI can help assist radiologists in identifying pathology such as pulmonary nodules’
- ‘May aid in characterising  pathology such as benign/malignant’
- ‘May aid in prioritising follow up scans for patients
- ‘Increase efficiency of workflow’...
- ‘AI will be used as a validation tool and as a tool to potentially discover hidden information that might have been overlooked’
- ‘It is unlikely that AI will replace radiologists in the near or distant future’
- ‘ We expect AI to evolve into a valuable educational resource’
- ‘Al may reduce errors’
- ‘Radiologists will emerge as critical elements in the AI training process’
- ‘AI may improve image quality and decrease radiation dose to patient’
- ‘May optimise MRI/CT scanner utilisation, reducing costs’
- ‘Shorten reporting time by pre-screening images and red flag for reporting’
- ‘Job satisfaction will increase’

Negative viewpoints:

- ‘Efficiency gained by AI will lead to fewer radiologists needed’...
- ‘AI will decrease job opportunities’...
- ‘AI applications will make the job profile more technical’...
- ‘Radiologists who use AI will replace radiologists who don’t’...
- ‘AI will make the radiologist-patient interaction more impersonal’...
- ‘Competition for registrar training positions will decrease over time’...
- ‘Medicine will not need radiologists at all’

Balanced:

- ‘The radiologist’s profile will remain unchanged’
- ‘The rate of dedication to sub specialities will remain unchanged’
- Includes articles that mention both positive and negative aspects in a balanced way
- Includes papers in journals that present AI & Radiology data objectively

Neutral:

- Includes articles that state facts or cannot be adequately defined into one of the other three categories

# appendix 4.1

## Tabulated summary of number of overall viewpoints presented by each author group and reflecting percentages for google search

| n=230 | Radiologist |  | Journalist |  | Doctor (Non-radiology) |  | Radiographer |  | Other |  |
| --- | --- | --- | --- | --- | --- | --- | --- | --- | --- | --- |
|  | n=89 | 38.70% | n=46 | 20.00% | n=16 | 6.96% | n=11 | 4.78% | n=68 | 29.57% |
|  |  |  |  |  |  |  |  |  |  |  |
| Positive | 41 | 46.07% | 24 | 52.17% | 3 | 18.75% | 5 | 45.45% | 26 | 38.24% |
| Negative | 4 | 4.49% | 1 | 2.17% | 0 | 0.00% | 0 | 0.00% | 3 | 4.41% |
| Balanced | 32 | 35.96% | 5 | 10.87% | 8 | 50.00% | 5 | 45.45% | 27 | 39.71% |
| Neutral | 12 | 13.48% | 16 | 34.78% | 5 | 31.25% | 1 | 9.09% | 12 | 17.65% |

# appendix 4.2.

## Tabulated summary of number of overall viewpoints presented by each author group and reflecting percentages for live feed

| N=151 | Radiologist |  | Journalist |  | Doctor (Non-radiology) |  | Commercial |  | Researcher |  | Other |  |
| --- | --- | --- | --- | --- | --- | --- | --- | --- | --- | --- | --- | --- |
|  | n=6 | 3.97% | n=100 | 66.23% | n=5 | 3.31% | n=19 | 12.58% | n=6 | 3.97% | n=15 | 9.93% |
|  |  |  |  |  |  |  |  |  |  |  |  |  |
| Positive | 3 | 50.00% | 64 | 64.00% | 3 | 60.00% | 16 | 84.21% | 1 | 16.67% | 8 | 53.33% |
| Negative | 0 | 0.00% | 1 | 1.00% | 0 | 0.00% | 0 | 0.00% | 0 | 0.00% | 1 | 6.67% |
| Balanced | 1 | 16.67% | 8 | 8.00% | 2 | 40.00% | 1 | 5.26% | 4 | 66.67% | 2 | 13.33% |
| Neutral | 2 | 33.33% | 27 | 27.00% | 0 | 0.00% | 2 | 10.53% | 1 | 16.67% | 4 | 26.67% |

# Appendix 3.1

## Media Viewpoints from Google Static Search (n=75, 29%)

| Media Source | Theme | Number |
| --- | --- | --- |
| Online Accessible Magazine | Diagnostic imaging | 5 |
|  | Radiology | 8 |
|  | Cardiology | 1 |
|  | Healthcare management | 6 |
| Website | Healthcare imaging technology | 24 |
|  | Healthcare | 2 |
| News | Corporate | 1 |
|  | Healthcare | 8 |
| Journal | Media | 5 |
| Blog |  | 10 |
| Forum | Healthcare | 4 |
| Social | Youtube | 1 |

# Appendix 3.2

## Specific commercial Website (n=32, 12%)

| Company | Number | Article Origin | Type of Article | Viewpoint |
| --- | --- | --- | --- | --- |
| Quantib | 1 | Marketing | Educational | Balanced |
| AIDOC | 1 | Marketing | Promotional | Positive |
| Hitachi | 1 | Research | Review | Balanced |
| Philips | 1 | Executive | Informational | Positive |
| Emerj | 5 | Journalist (2)  Operations (3) | Review (2)  Current applications (1)  Promotional (2) | Positive (2)  Positive (1)  Positive (2) |
| Siemens | 1 | Marketing | Promotional | Balanced |
| AGFA | 5 | Information (2)  Marketing (3) | Informational (2)  Promotional (3) | Positive (2)  Positive (1)  Neutral (2) |
| LUNIT | 1 | Research | Informational | Neutral |
| Intellias | 2 | Marketing  Marketing | Promotional  Promotional | Positive  Positive |
| Sectra | 1 | Operations | Informational | Positive |
| Harvard | 1 | Unknown | Promotional | Neutral |
| Predictive Analysis | 3 | Marketing (3) | Promotional (3) | Positive (3) |
| Missinglink.ai | 2 | Software engineer  Unknown | Informational  Instructive | Positive  Positive |
| Nanox | 1 | Unknown | Promotional | Positive |
| MDDI | 1 | Analytics | Informational | Balanced |
| Nanonets | 2 | Marketing (2) | Promotional (2) | Neutral (2) |
| Mindy | 1 | Radiologist | Interview | Positive |
| Signify | 1 | Principal analyst | Speculative | Positive |
| Fraunhofer Mevis | 1 | Unknown | Promotional | Neutral |
